# Safety and Immunogenicity of a Recombinant Tetanus Vaccine in Healthy Adults in China: A Randomized, Double‐Blind, Dose Escalation, Placebo‐ and Positive‐Controlled, Phase 1/2 Trial

**DOI:** 10.1002/advs.202002751

**Published:** 2021-06-03

**Authors:** Xiaowei Xu, Rui Yu, Lanlan Xiao, Jie Wang, Meihong Yu, Junjie Xu, Yajun Tan, Xiao Ma, Xiaoxin Wu, Jiangshan Lian, Kaizhou Huang, Xiaoxi Ouyang, Sheng Bi, Shipo Wu, Xiaoyan Wang, Jiandi Jin, Ling Yu, Huafen Zhang, Qi Wei, Jinfa Shi, Wei Chen, Lanjuan Li

**Affiliations:** ^1^ State Key Laboratory for Diagnosis and Treatment of Infectious Diseases National Clinical Research Centre for Infectious Diseases Collaborative Innovation Centre for Diagnosis and Treatment of Infectious Diseases First Affiliated Hospital College of Medicine Zhejiang University Hangzhou 310003 China; ^2^ Beijing Institute of Biotechnology Beijing 100071 China; ^3^ National Institutes for Food and Drug Control Beijing 102629 China; ^4^ Sichuan Zihao Times Pharmaceutical Co., Ltd Meishan Sichuan Province 610000 China

**Keywords:** recombinant tetanus vaccine, tetanus, tetanus toxoid vaccine

## Abstract

Tetanus is a fatal but vaccine‐preventable disease. The currently available tetanus vaccines are tetanus toxoid (TT)‐based. Although these vaccines are generally effective, challenges in vaccine development and access remain. A randomized, double‐blind, dose escalation, placebo‐ and positive‐controlled, phase 1/2 trial (ChiCTR1800015865) is performed to evaluate the safety and immunogenicity of an alternative recombinant tetanus vaccine based on the Hc domain of tetanus neurotoxin (TeNT‐Hc) in healthy adult volunteers. The primary outcome is the safety profile of the recombinant tetanus vaccine, and immunogenicity is the secondary outcome. 150 eligible participants were enrolled and randomly assigned to receive one of the three doses of recombinant tetanus vaccine (TeNT‐Hc 10/20/30 µg), TT vaccine, or placebo. The recombinant tetanus vaccine shows a good safety profile. The frequency of any solicited and unsolicited adverse events after each vaccination does not differ across the vaccine and placebo recipients. No serious treatment‐related adverse events occur. The recombinant tetanus vaccine shows strong immune responses (seroconversion rates, geometric mean titer, and antigen‐specific CD4+/CD8+ T‐cell responses), which are roughly comparable to those of the TT vaccine. In conclusion, the findings from this study support that recombinant tetanus vaccine is safe and immunogenic; thereby, it represents a novel vaccine candidate against tetanus.

## Graphical abstract

This study is the first randomized, phase 1/2 trial comparing dose escalation recombinant tetanus vaccine (TeNT‐Hc) with TT vaccine and placebo in healthy adults. All three doses of recombinant tetanus vaccine show a good safety profile, and strong immune responses, which are roughly comparable with TT vaccine. Thus, recombinant tetanus vaccine might be a novel vaccine candidate against tetanus.

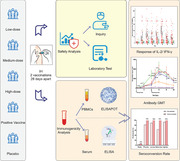

## 1 Introduction

Tetanus is a serious, often fatal, bacterial infection caused by tetanus neurotoxin (TeNT) produced by the anaerobic bacterium *Clostridium tetani*.^[^ ^1^ ^]^ In developed countries, tetanus cases are occasionally reported, most often among the older population (aged ≥60 years) or injection drug users. However, in low‐ and middle‐income countries, tetanus mortality rates remain high.^[^ ^2^, ^3^ ^]^ According to the Global Burden of Disease Study, an estimated 56 000 deaths annually were reported from tetanus worldwide in 2015.^[^ ^4^ ^]^ Treatment of tetanus involves providing supportive care, neutralizing tetanus toxoid (TT) antibodies, and eradicating bacteria at the wound site.^[^ ^5^ ^]^ In the absence of medical intervention, the case fatality rate of tetanus remains extremely high (near 100%), even in resource‐abundant regions.^[^ ^4^, ^6^ ^]^ Fortunately, tetanus is a vaccine‐preventable disease.^[^ ^1^, ^6^ ^]^ The TT vaccine was first developed in 1924 and since then has become part of the recommended immunization schedule for children owing to its effectiveness in protecting against tetanus.^[^ ^7^, ^8^ ^]^ By 2018, nearly 85% of infants worldwide (116.3 million infants) received three doses of diphtheria‐tetanus‐pertussis (DTP3) vaccine.^[^ ^9^ ^]^ However, DTP3 immunity is short‐lived in many individuals.^[^ ^1^ ^]^ Global tetanus cases and deaths have dramatically declined within the past two decades due to the scale up of immunization programs.^[^ ^10^, ^11^ ^]^ However, to maintain an adequate concentration of protection, additional booster doses should be administered every 10 years, especially in high‐risk populations, including women of reproductive age, soldiers, the older population, or injection drug users.^[^ ^1^, ^12^ ^]^ Barriers to access of the tetanus vaccine, especially monovalent tetanus vaccine, is one of many challenges in achieving universal coverage of the tetanus vaccine. Major impediments remain regarding the production of the current TT vaccine, including environmental pollution caused by during the formaldehyde detoxification process and low‐profit earnings, leading to low yields.^[^ ^13^ ^]^ Moreover, the quality of TT and how it is stored are important.^[^ ^7^ ^]^ Evidence has shown that 15 TT lots, in use from eight manufacturers in seven countries, had potency values below World Health Organization (WHO) requirements.^[^ ^14^ ^]^ These challenges, among others, demonstrate that ease and standardization of tetanus vaccine preparation are needed.

Advancements in vaccine development have been ongoing since the beginning of the genetic engineering revolution.^[^ ^15^ ^]^ With the advent of recombinant technologies, recombinant vaccines are being seen as promising solutions by the scientific community because of their relatively low production cost and innate stability for storage and transportation purposes.^[^ ^16^, ^17^ ^]^ The recombinant tetanus vaccine mainly consists of the Hc domain of TeNT (TeNT‐Hc), which possesses high immunogenicity and full binding affinity to neuronal cells via gangliosides in lipid rafts.^[^ ^4^, ^18^, ^19^ ^]^ This first‐in‐humans trial aims to assess the safety and immunogenicity of recombinant tetanus vaccine in a dose range of 10–30 µg. The primary focus of this study is to prevent tetanus; further, the investigational recombinant tetanus vaccine is a recombinant TeNT‐Hc‐based candidate vaccine that may be developed into a novel vaccine against tetanus.

## 2 Results

### 2.1 Vaccines and Placebo

The recombinant tetanus vaccine is composed of the Hc domain of TeNT—the recombinant protein TeNT‐Hc based on a patented technology (Chinese invention patent No: ZL2009101359720). All three doses of the recombinant tetanus vaccine and placebo were qualified by the China Institutes for Food and Drug Control. The inspection items included appearance, volume, identification test, osmotic pressure molar concentration, protein concentration, aluminum hydroxide concentration, sterility test, abnormal toxicity test, endotoxin test, and vaccine efficacy test. The placebo contains all the recombinant tetanus vaccine excipients but without any TeNT‐Hc. The recombinant tetanus vaccine and placebo were produced by the Beijing Institute of Biotechnology and Sichuan Zihao Times Pharmaceutical Co., Ltd. The TT vaccine used in the study was a monovalent adsorbed TT vaccine made by Olymvax Biopharmaceuticals (Chengdu, China).

### 2.2 Demographics

**Figure 1:**
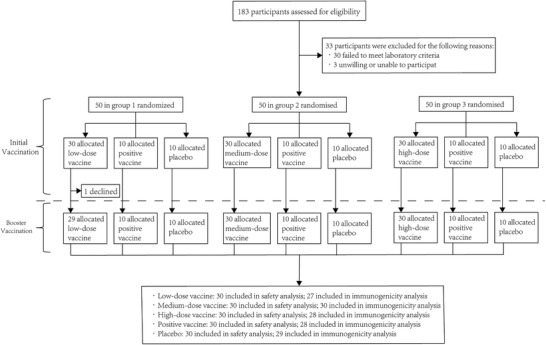
Trial profiles of the initial study and the booster study. TT = tetanus toxoid.

During the period between May 10, 2018, and June 17, 2018, 183 participants were screened for eligibility, of which 33 were excluded due to failure to meet laboratory criteria (*n* = 30) or withdrawal of consent (*n* = 3). A total of 150 participants received either a low‐dose recombinant tetanus vaccine, medium‐dose recombinant tetanus vaccine, high‐dose recombinant tetanus vaccine, TT vaccine, or placebo as prime immunization on day 0, with 30 participants in each treatment group. A total of 149 participants received booster immunizations. In this trial, 8 (5.3%) participants discontinued or were excluded from the per‐protocol population—three from the low‐dose group, two from the high‐dose group, two from the TT vaccine group, and one from the placebo group. A total of 150 participants were included in the safety analysis, and 142 (94.7%) participants in the immunogenicity analysis (**Figure** **1**). Baseline characteristics were similar between the groups (**Table** **1**).

**Table 1:** Demographic and baseline characteristics

Recombinant tetanus vaccine						
	Placebo	TT vaccine	Low‐dose	Medium‐dose	High‐dose	All doses combined
	(*N* = 30)	(*N* = 30)	(*N* = 30)	(*N* = 30)	(*N* = 30)	(*N* = 90)
**Sex**						
Male, *n* [%]	15 (50.0)	16 (53.3)	20 (66.7)	16 (53.3)	12 (40.0)	48 (53.3)
Female, *n* [%]	15 (50.0)	14 (46.7)	10 (33.3)	14 (46.7)	18 (60.0)	42 (46.7)
**Age [years]**						
Mean (SD)	28.3 ± 7.46	32.6 ± 10.62	27.4 ± 6.34	31.0 ± 9.40	26.1 ± 6.06	28.1 ± 7.63
Median (Q1, Q3)	25.1 (24.0, 30.6)	28.3 (23.7, 41.3)	25.1 (24.2, 26.7)	26.3 (23.8, 34.4)	24.8 (23.7, 25.8)	25.1 (23.9, 28.8)
Min, Max	(20, 47)	(19, 55)	(23, 54)	(22, 53)	(22, 56)	(22, 56)
**BMI [kg m^−^²] Mean (SD)**	23.00 ± 2.595	23.25 ± 2.675	22.39 ± 2.003	22.19 ± 1.993	21.75 ± 2.058	22.11 ± 2.013

TT = tetanus toxoid.

### 2.3 Reactogenicity and Safety

Our results suggest that all three doses of recombinant tetanus vaccine were highly safe for the healthy adult participants in China. Some of the subjects experienced solicited local and general reactions of mild or moderate intensity (Table 1). The occurrence of solicited adverse reactions within 0–7 days after each dose were not significantly different between all groups (*p* = 0.68, *p* = 0.50). Among the 90 participants who received the recombinant tetanus vaccine, 32 (35.6%) reported at least one solicited adverse reaction within the first 7 days after the initial vaccination, and 42 (47.2%) reported after booster vaccination. The injection‐site reactions were frequent after both doses, with redness (19 [21.0%], 21 [23.6%]) most commonly reported, followed by injection‐site pain without touching (17 [19.0%], 22 [24.7%]), induration (10 [11.0%], 19 [21.3%]), itching (5 [6.0%], 6 [6.7%]), and rash (1 [1.0%], 0 [0%]). Systemic adverse reactions are typically rare, and none have been reported. Redness was the only injection‐site adverse reaction reported more frequently in the medium‐dose (*p* = 0.02) and high‐dose (*p* = 0.01) recombinant tetanus vaccine groups than in the TT vaccine groups. However, increased rates of injection‐site reactions were not associated with increases in TeNT‐Hc dose, and increases in the severity of local reactions were not linked to booster vaccinations. At the end of the study, all local reactions were completely resolved. None of the injection‐site reactions required any medical treatment.

The occurrence of any unsolicited adverse events (*p* = 0.26, *p* = 0.79) on day 28 after each vaccination did not differ across the vaccine and placebo recipients. In the recombinant tetanus vaccine group, mild (46 [51.0%]) and moderate (21 [23.0%]) unsolicited adverse events were reported most frequently; however, no significant differences were found between the recombinant tetanus vaccine and placebo groups (*p* = 0.138 and *p* = 0.243, respectively).

Clinically relevant abnormalities in vital signs or laboratory safety parameters were not observed. Laboratory adverse events were detected in 12 participants on day 7 after the prime vaccination, but all were clinically insignificant. The most common adverse events were hyperuricemia (*n* = 7 [58.3%]), followed by high creatine kinase (*n* = 3 [25.0%]) and hypertransaminemia (*n* = 1 [8.3%], **Table** **2**). Although six serious adverse events were reported in five participants during the study period (Supporting Information), all serious adverse events were deemed unrelated to the vaccination.

**Table 2:** Participants reporting reactogenicity signs or symptoms following administration of the first dose and second dose

Recombinant tetanus vaccine							
	Placebo	TT vaccine	Low‐dose	Medium‐dose	High‐dose	All doses combined	
	(*N* = 30)	(*N* = 30	(*N* = 30)	(*N* = 30)	(*N* = 30)	(*N* = 90)	*p* value*
**Solicited adverse reactions within 0–7 days**							
First dose	13/30 (43.3)	10/30 (33.3)	8/30 (26.7)	12/30 (40.0)	12/30 (40.0)	32/90 (35.6)	0.6814
Second dose	10/30 (33.3)	17/30 (56.7)	14/29 (48.3)	14/30 (46.7)	14/30 (46.7)	42/89 (47.2)	0.4973
**Injection‐site adverse reactions within 0–7 days**							
**Pain**							
First dose	8/30 (26.7)	6/30 (20)	4/30 (13.3)	7/30 (23.3)	6/30 (20)	17/90 (18.9)	0.7745
Second dose	5/30 (16.7)	5/30 (16.7)	10/29 (34.5)	6/30 (20)	6/30 (20)	22/89 (24.7)	0.4324
**Induration**							
First dose	7/30 (23.3)	1/30 (3.3)	2/30 (6.7)	5/30 (16.7)	3/30 (10)	10/90 (11.1)	0.1475
Second dose	3/30 (10)	8/30 (27)	6/29 (20.7)	9/30 (30)	4/30 (13.3(	19/89 (21.3)	0.249
**Redness**							
First dose	6/30 (20)	2/30 (6.7)	0/30 (0)	9/30 (30)	10/30 (33.3)	19/90 (21.1)	0.2562
Second dose	7/30 (23.3)	2/30 (6.7)	5/29 (17.2)	8/30 (26.7)	8/30 (26.7)	21/89 (23.6)	0.2562
**Itch**							
First dose	1/30 (3.3)	1/30 (3.3)	0/30 (0)	2/30 (6.7)	3/30 (10)	5/90 (5.6)	0.6045
Second dose	0/30 (0)	1/30 (3.3)	0/29 (0)	3/30 (10)	3/30 (10)	6/89 (6.7)	0.1511
**Rash**							
First dose	0/30 (0)	0/30 (0)	0/30 (0)	0/30 (0)	1/30 (3.3)	1/90 (1.1)	1
Second dose	0/30 (0)	0/29 (0)	0/29 (0)	0/29 (0)	0/30 (0)	0/89 (0)	1
**Systemic adverse reactions within 0–7 days**							
**Fever**							
First dose	0/30 (0)	0/30 (0)	0/30 (0)	0/30 (0)	0/30 (0)	0/90 (0)	1
Second dose	0/30 (0)	0/30 (0)	1/29 (3.4)	0/30 (0)	0/30 (0)	0/89 (0)	1
**Fatigue**							1
First dose	0/30 (0)	0/30 (0)	0/30 (0)	0/30 (0)	0/30 (0)	0/90 (0)	1
Second dose	1/30 (3.3)	0/30 (0)	0/29 (0)	0/30 (0)	0/30 (0)	0/89 (0)	
**Nausea**							
First dose	0/30 (0)	0/30(0)	0/30 (0)	1/30 (3.3)	0/30 (0)	1/90 (1.1)	1
Second dose	0/30 (0)	0/30(0)	0/29 (0)	0/30 (0)	0/30 (0)	0/89 (0)	1
**Cough**							
First dose	0/30 (0)	1/30 (3.3)	0/30 (0)	0/30 (0)	0/30 (0)	0/90 (0)	1
Second dose	0/30 (0)	1/30 (3.3)	0/29 (0)	0/30 (0)	0/30 (0)	0/89 (0)	1
**Pain**							
First dose	0/30 (0)	0/30 (0)	1/30 (3)	1/30 (3.3)	0/30 (0)	2/90 (2.2)	1
Second dose	1/30 (3.3)	0/30 (0)	0/29 (0)	1/30 (3.3)	0/30 (0)	1/89 (1.1)	1
**Pharyngeal diseases**							
First dose	0/30 (0)	1/30 (3.3)	0/30 (0)	0/30 (0)	0/30 (0)	0/90 (0)	1
Second dose	0/30 (0)	0/30 (0)	0/29 (0)	0/30 (0)	1/30 (3.3)	1/89 (1.1)	1
**Headache**							
First dose	0/30 (0)	1/30 (3.3)	0/30 (0)	0/30 (0)	0/30 (0)	0/90 (0)	1
Second dose	0/30 (0)	0/30 (0)	0/29 (0)	0/30 (0)	0/30 (0)	0/89 (0)	1
**Unsolicited adverse events within 0–28 days**							
First dose	26/30 (86.7)	22/30 (73.3)	27/30 (90.0)	27/30 (90.0)	23/30 (76.7)	77/90 (85.6)	0.2598
Second dose	23/30 (76.7)	23/30 (76.7)	22/29 (75.9)	20/30 (66.7)	20/30 (66.7)	62/89 (69.7)	0.7889
**Adverse events within 0–28 days**							
First dose	13/30 (43.3)	12/30 (40.0)	8/30 (26.7)	12/30 (40.0)	13/30 (43.3)	33/90 (36.7)	0.6595
Second dose	10/30 (33.3)	17/30 (56.7)	16/29 (55.2)	15/30 (50.0)	14/30 (46.7)	45/89 (50.6)	0.3864
**Laboratory abnormalities within 0–7 days**							
First dose	4/30 (13.3)	8/30 (26.7)	4/30 (13.3)	0/30 (0)	0/30 (0)	4/90 (4.4)	0.001
Second dose	7/30 (76.7)	6/30 (76.7)	8/29 (27.5)	0/30 (0)	0/30 (0)	8/89 (8.9)	<0.0001

Data are presented as n (%). *p values were generated from comparisons across five groups. TT, tetanus toxoid.

### 2.4 Humoral Immune Response

**Figure 2:**
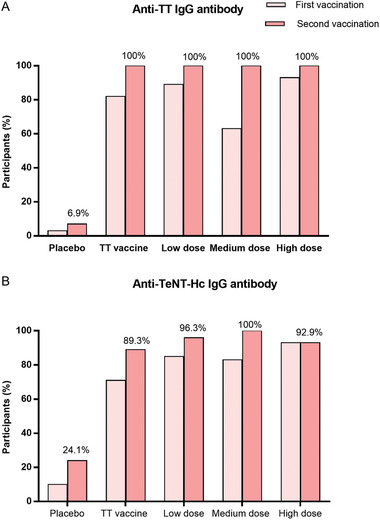
Proportion of seroconversion of anti‐TT IgG or anti‐TeNT‐Hc IgG antibodies 28 days after each vaccination (A). Proportion of seroconversion of anti‐TT IgG antibody (B). Proportion of seroconversion of anti‐TeNT‐Hc IgG antibody. Seroconversion, defined as a four‐time rise in antibody titer compared with baseline titer (baseline titer ≥50), or titer ≥ 200 (baseline titer <50), 4 weeks after each vaccination. TT = tetanus toxoid.

Across all recombinant tetanus vaccine groups, the seroconversion rates of anti‐TT and anti‐TeNT‐Hc immunoglobulin G (IgG) antibodies reached 81.2% (95% CI: 0.727–0.897)/87.1% (95% CI: 0.798–0.943) after the first immunization (day 28), increased to 100% (95% CI: 1.00–1.00)/96.5% (95% CI: 0.925–1.00) after the second immunization (day 56), and remained at a high level (97.6%/92.9%) for 6 months after the second immunization (day 208). Participants who received the medium‐dose recombinant tetanus vaccine reported a lower seroconversion rate of anti‐TT antibody 28 days after prime vaccination than those who received the TT vaccine (63.3%:92.9%, *p* = 0.018, Table S4, Supporting Information). However, the seroconversion rates of anti‐TeNT‐Hc antibody were significantly higher in the high‐dose group (92.9%:71.4%, *p* = 0.022) at day 28 and in the low‐dose (96.3%:64.3%, *p* = 0.02) and medium‐dose groups (96.7%:64.3%, *p* = 0.01) at day 208 than in the TT vaccine group (**Figures** **2**A, B and Table S5, Supporting Information).

**Figure 3:**
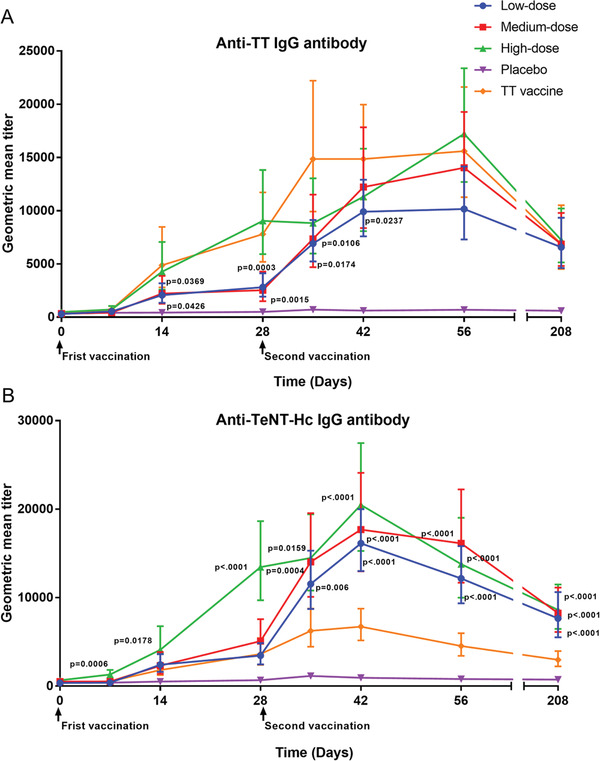
Geometric mean titer of anti‐TT or anti‐TeNT‐Hc IgG antibody, after initial and booster vaccination (A). Geometric mean titer of anti‐TT IgG antibody (B). Geometric mean titer of anti‐TeNT‐Hc IgG antibody. *p* values were generated by comparing the positive vaccine and all doses of the recombinant tetanus vaccine, the data were compared using Wilcoxon rank sum test. TT = tetanus toxoid.

After prime vaccination, the geometric mean titer (GMT) of anti‐TT and anti‐TeNT‐Hc IgG antibodies increased rapidly in both the recombinant tetanus vaccine and TT vaccine groups (**Figures** **3**A and B). For participants immunized with recombinant tetanus vaccine, the GMT of anti‐TT IgG antibody increased sevenfold (*p* < 0.001) after the first vaccination, from 358.7 (95% CI: 304.8–422.2) to 3988.1 (95% CI: 3014.5–5276.3) and 38‐fold to 13551.9 (95% CI: 11297.3–16256.4) after the second vaccination (Table S6, Supporting Information). Meanwhile, the GMT of TeNT‐Hc IgG antibody increased 12‐fold (*p* < 0.001) after the first vaccination, from 493.0 (95% CI: 395.6–614.3) to 6194.6 (95% CI: 4902.1–7828.0) and 28‐fold to 13887.5 (95% CI: 11696.2–16489.4) after the second vaccination (Table S7, Supporting Information).

Compared to the TT vaccine group, the GMT of anti‐TT IgG antibodies was lower in the low‐dose group at 14, 28, 35, and 42 days and in the medium‐dose group at 14, 28, and 35 days after the first vaccination. However, the high‐dose group showed higher GMT of anti‐TeNT‐Hc IgG antibodies than the TT vaccine group at 7, 14, and 28 days after the first vaccination (*p* < 0.05, Figure 3A), and all recombinant tetanus vaccine groups showed higher GMT of anti‐TeNT‐Hc IgG antibodies at 35, 42, 56, and 208 days after the first vaccination (*p* < 0.05, Figure 3B).

### 2.5 Cell‐Mediated Immune Response

**Figure 4:**
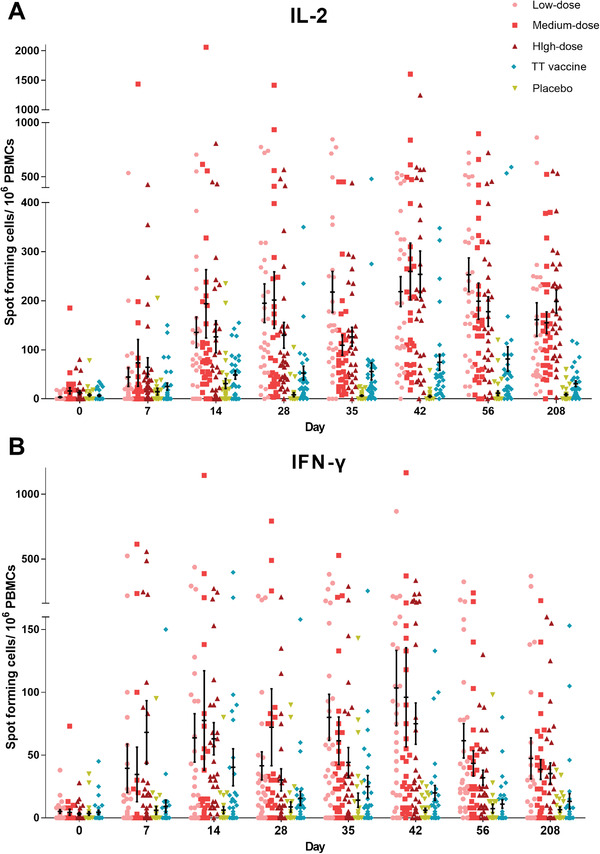
Specific T‐cell response measured by enzyme‐linked immunosorbent spot (ELISPOT) assay at different time points before and after prime and booster vaccination. A) IL‐2 expressing T‐cells, number of ELISPOT spot forming cells (SFC)/10⁶ cells. B) IFN‐*γ* expressing T cells, number of ELISPOT SFC/10⁶ cells. TT = tetanus toxoid.

The specific T‐cell responses quantified by interleukin‐2 (IL‐2) and interferon‐*γ* (IFN‐*γ*) showed moderate increases after prime vaccination, with 115.0/20.0 spot‐forming cells (IQR 65.0–218.0/3.0–35.0) in the low‐dose group, 80.5/11.5 spot‐forming cells (30.0–245.0/0–35.0) in the medium‐dose group, and 70.0/10.0 spot‐forming cells (40.0–141.5/1.5–30.0) in the high‐dose group; these were significantly higher than those in the TT group ( 33.0/8.0 spot‐forming cells [9.0–63.0/0–16.5]） and placebo group（0/0 spot‐forming cells [0‐3.0/0–3.0]） (Tables S10 and S11, Supporting Information). Booster vaccination with recombinant tetanus vaccine elicited stronger immune responses in specific T‐cells from the TeNT‐Hc protein. Positive T‐cell responses among specific CD4 and CD8 T‐cells in participants receiving recombinant tetanus vaccine peaked at day 14 after booster vaccination and then declined gradually for the remainder of the study (**Figure** **4**A,B).

The recombinant tetanus vaccine group reported greater expression of IL‐2 and IFN‐*γ* by T‐cells than the TT vaccine group. IL‐2 responses in the low‐dose and high‐dose groups were higher than those in the TT vaccine group at 14 and 35 days after the first vaccination. All three doses of recombinant tetanus vaccine showed a higher response for IL‐2 than the TT vaccine group at days 28, 42, 56, and 208 after the first vaccination. The responses for IFN‐*γ* were higher in the low‐dose group at days 35 and 42 and in the high‐dose group at days 7 and 42 than those in the TT vaccine group. In addition, all three vaccine doses elicited greater IFN‐*γ* expression by T‐cells than the TT vaccine group at day 28 as well as 6 months after the second vaccination (Figure 4A,B).

## 3 Discussion

This is the first randomized trial to assess the safety and immunogenicity of a recombinant tetanus vaccine versus the traditional TT vaccine in healthy adults. In this trial, the recombinant tetanus vaccine was found to be safe, with no significant differences in adverse reactions compared to that with placebo and no serious treatment‐related adverse events. The proportion of seroconversion responders at 4 weeks after each vaccination and 6 months of follow‐up after completion did not differ significantly between the recombinant tetanus vaccine and TT vaccine groups. At most of the visit time points, the two vaccines demonstrated similar immunogenicity. The recombinant tetanus vaccines tended to induce more anti‐TeNT‐Hc IgG antibodies than the TT vaccine, while the TT vaccine tended to induce more anti‐TT IgG antibodies than the recombinant tetanus vaccine. Our trial revealed that both recombinant tetanus vaccine and TT vaccine were highly immunogenic and elicited high antibody responses with immunogenicity for a prolonged duration. Assuming that the WHO suggested enzyme‐linked immunosorbent assay (ELISA) value of 0.1–0.2 IU/mL is the level of adequate protection for humans,^[^ ^20^ ^]^ all three doses of the recombinant tetanus vaccine were able to induce sufficiently high TT and TeNT‐Hc‐specific antibody responses (exceeding 0.2 IU/mL, Tables S8 and S9, Supporting Information). A large number of cases of tetanus continues to be reported in internationally published literature, indicating that tetanus remains a major problem, particularly among adults. Over recent decades, critical care facilities have improved in many low‐income and middle‐income countries, with a likely significant decline in mortality rates and little change in morbidity.^[^ ^1^ ^]^ However, tetanus infection does not confer immunity, and active vaccination is recommended to ensure continued protection.^[^ ^5^ ^]^ Our study revealed that one or two doses of vaccination with the recombinant tetanus vaccine may be sufficient to induce memory immune responses and produce antibodies against tetanus promptly and robustly several months or years later.

TeNT is a protein of about 150 kDa and consists of a 50 kDa light chain and a 100 kDa heavy chain.^[^ ^21^ ^]^ The heavy chain contains an N‐terminal translocation domain (HN) and a C‐terminal receptor binding domain (Hc).^[^ ^22^ ^]^ In this study, TT induced the production of antibodies against both TeNT‐Hc and TeNT‐HN as well as against the light chain. However, the antibodies induced by TT against TeNT‐Hc are the primary, protective neutralizing antibodies.^[^ ^23^, ^24^ ^]^ The recombinant tetanus vaccine is mainly composed of the TeNT‐Hc protein, which is expressed as a soluble protein with appropriate folding and high yields.^[^ ^25^, ^26^ ^]^ The bioengineering characteristics of TeNT‐Hc, including its ability to retain full binding affinity to neurons and induce a protective immunological response against the full‐length holotoxin, are described elsewhere.^[^ ^4^, ^18^, ^19^ ^]^ Another paper reported that TeNT‐Hc can induce equine tetanus immunoglobulin similar to TT as an immunogen in horses.^[^ ^26^ ^]^ Moreover, TeNT‐Hc can be used as a carrier protein for polysaccharide vaccines like TT to induce immune memory.^[^ ^27^ ^]^ TeNT‐Hc has previously been cloned and expressed with several other vaccine platforms.^[^ ^28^, ^29^ ^]^ Several studies succeeded in producing a high expression of TeNT‐Hc; however, they failed in reaching productization due to poor efficacy compared with the commercial TT vaccine.^[^ ^30^, ^31^ ^]^ The recombinant tetanus vaccine in this trial was based on studies on the soluble protein TeNT‐Hc and our previous patent, which was the first report on the purification of a non‐tag TeNT‐Hc isoform from *E. coli* in large quantities for potential human application.^[^ ^25^, ^26^, ^27^, ^32^ ^]^

Vaccine efficacy is based on whether the host immune response against an antigen can elicit a memory T‐cell response over time. Once infection occurs, T‐cells transform into a group of effector T‐cells that can quickly acquire and expand the effector function after antigen re‐exposure, ultimately surviving long‐term and providing continual immunity.^[^ ^33^ ^]^ In this study, we found that the recombinant tetanus vaccine induced significantly more specific CD4+ T‐cells than the TT vaccine, especially in the IFN‐*γ* T‐cell response. Altogether, our data further indicate that recombinant tetanus vaccine may be a suitable vaccine candidate, given the differences in special cell‐mediated immunity between the recombinant tetanus vaccine and TT vaccine.

Previous papers have reported that TT vaccine is effective in protecting against tetanus, with a failure rate of 4/100 million immunocompetent people. Further, reactions to the TT vaccine are estimated to be 1 in 50 000 injections, and most reactions are not severe; local tenderness, edema, flu‐like illness, and low‐grade fever are the most often encountered reactions.^[^ ^16^ ^]^ In our study, the recombinant tetanus vaccine showed equal safety and immunizing potency as the TT vaccine. Conventional vaccines (inactivated vaccine and attenuated vaccine) require a mass culture of associated or related organisms and long incubation periods; further, special requirements during storage and transportation add to the cost of vaccine preparation.^[^ ^34^ ^]^ With advances in technology, safe, cheap, and effective vaccine candidates have been produced, and the field began to focus on the development of recombinantly expressed antigens known as subunit vaccines.^[^ ^35^ ^]^ The recombinant vaccine is a subunit vaccine that uses recombinant proteins from pathogens. This implies that once the proteins having the capacity to vaccinate have been identified, they can be produced in sufficient quantity at a low cost.^[^ ^16^ ^]^ For these reasons, recombinant tetanus vaccine can be used as a candidate vaccine against tetanus.

Although the data presented here suggest good safety and immunogenicity of the recombinant tetanus vaccine, results from this phase 1/2 trial in a small proportion of healthy adults from a single region in China could be limited in generalizability. A phase 3 study of the recombinant tetanus vaccine should be conducted in a large population across China. Meanwhile, the sample size of a phase 1/2 study limits the detection of all rare vaccine‐related adverse events. Extended trials will have to confirm the safety of recombinant tetanus vaccine and the persistence of functional antibodies in a larger, more heterogeneous population.

## 4 Conclusion

In summary, the present preliminary clinical trial results showed that the recombinant tetanus vaccine induced adequate protective neutralizing antibodies (anti‐TT and anti‐TeNT‐Hc antibodies) and specific T‐cell responses in healthy adults. No significant differences in adverse reactions were found between the recombinant tetanus vaccine and placebo groups, and no serious treatment‐related adverse events occurred. This trial revealed that recombinant tetanus vaccine might have the potential to serve as a safe and effective vaccine against tetanus.

## 5 Experimental Section

### Study Design and Participants

The authors conducted this randomized, double‐blind, dose escalation, placebo‐ and positive‐controlled, phase 1/2 trial in Hangzhou City, Zhejiang Province, China, from May 10, 2018, to December 31, 2018. A total of 150 healthy adults of both sexes, aged 18–60 years, meeting all eligibility criteria were enrolled. Two doses each of the recombinant tetanus vaccine, TT vaccine, and placebo were administered as intramuscular injections in the deltoid muscle at an interval of 28 days. The primary exclusion criterion was a previous history of TT or DTP vaccination within the past 5 years. Women who were pregnant, breastfeeding, or planning to become pregnant within the next 3 months were excluded. A complete list of the inclusion and exclusion criteria is provided in the Supporting Information. All participants provided written informed consent prior to the initiation of the study. The study protocol and its amendments were reviewed and approved by the Ethics Committee of the First Affiliated Hospital, Zhejiang University School of Medicine. The study was conducted in accordance with the Declaration of Helsinki and Good Clinical Practice guidelines. The trial was registered with Chictr.org under the identifier ChiCTR1800015865.

### Randomization and Masking

The eligible participants were sequentially enrolled in ascending dose groups of three stages, and participants in each stage were randomly assigned at a 3:1:1 ratio based on computer‐generated random numbers to receive one of the three doses of recombinant tetanus vaccine (TeNT‐Hc 10/20/30 µg, *n* = 30 per dose group), TT vaccine (*n* = 30, in three groups of 10 each), or placebo (*n* = 30, in three groups of 10 each). As the recombinant tetanus vaccine, TT vaccine, and placebo were clearly labeled, three unmasked study nurses were assigned to prepare all vaccines and placebo preparations. The study investigators, participants, and other team members were masked to the vaccination allocation throughout the study. An emergency unmasked process could be performed only on special requests from the study investigators or ethical committee if ethical and emergency clinical concerns were raised.

### Procedures

This study was performed in three stages (50 participants in each stage)—among the first 50 participants, 30 were randomized to low‐dose recombinant tetanus vaccine, and 10 each to TT vaccine or placebo after a blinded review showed no evidence of dose‐related reactogenicity or toxic effects within 7 days; the next 50 participants were randomized in the same ratio, but with 30 to the medium‐dose, and when this was similarly seen to be safe, the last 50 participants were randomized in the same ratio, now with 30 to the high‐dose (Figure 1). The second vaccination was administered 4 weeks after the first vaccination for all participants. After each administration, the subjects remained in the hospital and were observed by investigators for 30 min.

Laboratory assessments (including routine blood examination, coagulation function test, liver and renal function test, and routine urine test) and vital sign checks (heart rate, blood pressure, and ear temperature measurements) were performed at baseline and days after each vaccination for safety analysis. Participants were required to keep a diary for 56 days after the first vaccination to record any abnormalities (local pain without touching, induration, redness, itching, rash, and swelling) or general reactions and their severity (nausea, cough, muscle pain, and headache). Diaries were reviewed by a physician during each visit to verify the accuracy of the reported symptoms. Further, participants were interviewed for adverse events, and their responses were recorded by clinical research coordinators at each visit. The severity of clinical adverse events was graded on a three‐point scale from mild to moderate to severe, indicating the subjective intensity of the symptoms. The severity grading of adverse events was based on the standard guidelines issued by the China Food and Drug Administration.

Participant serological biomarkers, including TT and TeNT‐Hc indicators, and cellular immune assays comprised immunological specimens. We obtained specimens at baseline and at 7, 14, and 28 days after each vaccination and 6 months after the second vaccination for immunogenicity analysis. Antibody titers against TeNT‐Hc and TT were determined by IgG enzyme‐linked immunosorbent assay (ELISA) at baseline. An immune response was defined as seroconversion to a TeNT‐Hc or TT antibody titer >1:200 (baseline titer <1:50) or a fourfold titer increase (baseline titer ≥ 1:50) after one or two vaccinations. The antigen‐specific CD4+/CD8+ T‐cell responses were assessed using IFN‐*γ* and IL‐2 enzyme‐linked immunosorbent spot (ELISPOT) assays with peripheral blood mononuclear cells stimulated with recombinant TeNT‐Hc protein, and the duration of specific immune responses was assessed up to 6 months after the second vaccination.

### Outcomes

The primary outcome was the safety profile of two doses of recombinant tetanus vaccines in healthy adults aged 18–60 years, including occurrence of solicited adverse reactions within 7 days after each vaccination, solicited and unsolicited adverse events within 28 days after each vaccination, abnormalities in laboratory measures within 0–7 days after each vaccination, and any serious adverse events throughout the follow‐up period. The secondary outcomes were the immune responses measures by the percentage of seroconversion of anti‐TeNT‐Hc or anti‐TT antibody 28 days after each vaccination, the GMT of antibodies against TT and TeNT‐Hc, and the magnitude of antigen‐specific CD4+/CD8+ T‐cell responses.

### Statistical Analyses

Data transformation, normalization, and evaluation of outliers were not used in this study. Continuous variables presented as mean (± standard deviation) or median (interquartile range). Considering a 5% dropout rate, the authors estimated that a total of 150 participants were needed for this non‐inferiority trial. They considered the seroconversion rate of anti‐TT IgG for the traditional TT vaccine to be 90%. They assumed that it is adopted if the recombinant tetanus vaccine has a seroconversion rate of at least 80%. The actual difference in the seroconversion rate of anti‐TT IgG between the traditional TT vaccine and recombinant tetanus vaccine was set to ±5%. The randomization allocation ratio was set to 3:1:1 for the recombinant tetanus vaccine, traditional TT vaccine, and placebo groups. Comparisons between different groups were performed using *χ* ^2^ tests (or Fisher's exact test for smallest count <5) for categorical variables and analysis of variance (ANOVA) (or the Wilcoxon rank sum test for variables with non‐normal distribution) for continuous variables. Multiple comparisons were performed using the Bonferroni procedure when there was a significant difference across groups. Statistical analysis was performed using SAS software, version 9.4 (SAS Institute Inc., Cary, NC, US). Statistical significance was defined by *p* < 0.05 (two‐tailed). The safety endpoints were summarized in an intention‐to‐treat cohort that included all participants who received an injection. The immunological analysis was based on a per‐protocol cohort that included all participants who adhered to the study protocol and had no major protocol deviations.

## Conflict of Interest

The authors declare no conflict of interest.

## Author Contributions

X.X., R.Y., L.X., J.W., and M.Y. contributed equally. L.L. and X.X. are the principal investigators of this study. L.L., W.C., and R.Y. conceived the idea and designed the study. X.X., R.Y., and L.X. contributed to the study design, data interpretation, drafting of the manuscript, and revision of the report. J.X., Y.T., K.H., X.O., S.W., and X.M. contributed to the laboratory analyses, data interpretation, and revision of the report. J.W., M.Y., X.X., S.B., X.W., J.J., L.Y., and H.Z. participated in the site work, including recruitment, follow‐up, and data collection. R.J. contributed to vaccine management. R.Y., Q.W., and J.S. supervised the entire study process and took responsibility for all data from both sites and laboratories. R.Y. and L.X. contributed to the statistical analyses. All authors have reviewed and approved the final version of the manuscript.

## Supporting information

Supporting Information

## Data Availability

The data that support the findings of this study are available from the corresponding author upon reasonable request.

## References

[1] L. M. Yen , C. L Thwaites , Lancet 2019, 393, 1657. 30935736 10.1016/S0140-6736(18)33131-3

[2] N. J. Beeching , N. S Crowcroft , BMJ 2005, 330, 208. 15677636 10.1136/bmj.330.7485.208 PMC546055

[3] A. Filia , A. Bella , C. von Hunolstein , A. Pinto , G. Alfarone , S. Declich , M. C Rota , Vaccine 2014, 32, 639. 24370712 10.1016/j.vaccine.2013.12.012

[4] H. H. Kyu , J. E. Mumford , J. D. Stanaway , R. M. Barber , J. R. Hancock , T. Vos , C. J. Murray , M. Naghavi , BMC Public Health 2017, 17, 179. 28178973 10.1186/s12889-017-4111-4 PMC5299674

[5] C. Rodrigo , D. Fernando , S. Rajapakse , Crit. Care 2014, 18, 217. 25029486 10.1186/cc13797 PMC4057067

[6] The Weekly Epidemiological Record, 2017, p. 92.

[7] J. J. Farrar , L. M. Yen , T. Cook , N. Fairweather , N. Binh , J. Parry , C. M Parry , J. Neurol., Neurosurg. Psychiatry 2000, 69, 292. 10945801 10.1136/jnnp.69.3.292 PMC1737078

[8] N. Ruhrman‐Shahar , J. Torres‐Ruiz , P. Rotman‐Pikielny , Y. Levy , Immunol. Res. 2017, 65, 157. 27435706 10.1007/s12026-016-8822-x

[9] Imunization coverage, https://www.who.int/en/news‐room/fact‐sheets/detail/immunization‐coverage (accessed: July 15, 2020).

[10] A. A. Khan , A. Zahidie , F. Rabbani , BMC Public Health 2013, 13, 322. 23570611 10.1186/1471-2458-13-322 PMC3637612

[11] Eliminating maternal and neonatal tetanus, https://www.who.int/immunization/diseases/MNTE_initiative/en/ (accessed: July 15, 2020).

[12] G. Ramakrishnan , M. Wright , M. Alam , C. Naylor , M. Kabir , A. Zerin , T. Ferdous , K. Pedersen , B. J. Hennig , J. R. Donowitz , R. Wegmuller , R. Haque , W. A. Petri Jr. , J. Herbein , C. A. Gilchrist , Diagn. Microbiol. Infect. Dis. 2017, 87, 272. 27916543 10.1016/j.diagmicrobio.2016.11.011 PMC5292284

[13] J. F. Mosser , W. Gagne‐Maynard , P. C. Rao , A. Osgood‐Zimmerman , N. Fullman , N. Graetz , R. Burstein , R. L. Updike , P. Y. Liu , S. E. Ray , L. Earl , A. Deshpande , D. C. Casey , L. Dwyer‐Lindgren , E. A. Cromwell , D. M. Pigott , F. M. Shearer , H. J. Larson , D. J. Weiss , S. Bhatt , P. W. Gething , C. J. L. Murray , S. S. Lim , R. C. Reiner Jr. , S. I. Hay , Lancet 2019, 393, 1843. 30961907 10.1016/S0140-6736(19)30226-0 PMC6497987

[14] V. Dietz , J. B. Milstien , F. van Loon , S. Cochi , J. Bennett , Bull. W. H O. 1996, 74, 619. 9060223 PMC2486793

[15] S. Plotkin , Proc. Natl. Acad. Sci. USA 2014, 111, 12283. 25136134 10.1073/pnas.1400472111 PMC4151719

[16] M. J Francis , Vet. Clin. North Am.: Small Anim. Pract. 2018, 48, 231. 29217317 10.1016/j.cvsm.2017.10.002 PMC7132473

[17] J. Lee , S. A. Kumar , Y. Y. Jhan , C. J Bishop , Acta Biomater. 2018, 80, 31. 30172933 10.1016/j.actbio.2018.08.033 PMC7105045

[18] T. C. Umland , L. M. Wingert , S. Swaminathan , W. F. Furey , J. J. Schmidt , M. Sax , Nat. Struct. Mol. Biol. 1997, 4, 788. 10.1038/nsb1097-788 9334741

[19] T. C. Umland , L. Wingert , S. Swaminathan , J. J. Schmidt , M. Sax , Acta Crystallogr., Sect. D: Biol. Crystallogr. 1998, 54, 273. 9761892 10.1107/s0907444997009025

[20] WHO , Weekly Epidemiological Record, May 19 2006, 197.

[21] U. Eisel , W. Jarausch , K. Goretzki , A. Henschen , J. Engels , U. Weller , M. Hudel , E. Habermann , H. Niemann , EMBO J. 1986, 5, 2495. 3536478 10.1002/j.1460-2075.1986.tb04527.x PMC1167145

[22] M. Matsuda , M. Yoneda , Biochem. Biophys. Res. Commun. 1974, 57, 1257. 4208570 10.1016/0006-291x(74)90831-6

[23] G. Lalli , J. Herreros , S. L. Osborne , C. Montecucco , O. Rossetto , G. Schiavo , J. Cell Sci. 1999, 112, 2715. 10413679 10.1242/jcs.112.16.2715

[24] A. Rummel , S. Bade , J. Alves , H. Bigalke , T. Binz , J. Mol. Biol. 2003, 326, 835. 12581644 10.1016/s0022-2836(02)01403-1

[25] R. Yu , L. Hou , C. Yu , S. Liu , J. Ren , T. Fang , X. Zhang , W. Chen , Immunobiology 2011, 216, 485. 20888666 10.1016/j.imbio.2010.09.001

[26] R. Yu , C. Ji , J. Xu , D. Wang , T. Fang , Y. Jing , C. Kwang‐Fu Shen , W. Chen , Biomed Res. Int. 2018, 2018, 6057348. 30687751 10.1155/2018/6057348 PMC6330821

[27] R. Yu , J. Xu , T. Hu , W. Chen , Mediators Inflammation 2020, 2020, 9596129. 10.1155/2020/9596129 PMC7355367 32714092

[28] J. L. Halpern , W. H. Habig , E. A. Neale , S. Stibitz , Infect. Immun. 1990, 58, 1004. 2318526 10.1128/iai.58.4.1004-1009.1990 PMC258574

[29] N. F. Fairweather , V. A. Lyness , D. J Maskell , Infect. Immun. 1987, 55, 2541. 3312002 10.1128/iai.55.11.2541-2545.1987 PMC259939

[30] A. V. Ribas , P. L. Ho , M. M. Tanizaki , I. Raw , A. L. T. O. Nascimento , Biotechnol. Appl. Biochem. 2000, 31, 91. 10744952 10.1042/ba19990084

[31] A. J. Makoff , M. D Oxer , Nucleic Acids Res. 1991, 19, 2417. 2041779 10.1093/nar/19.9.2417 PMC329451

[32] R. Yu , T. Fang , S. Liu , X. Song , C. Yu , J. Li , L. Fu , L. Hou , J. Xu , W. Chen , Toxins 2016, 8, 194. 10.3390/toxins8070194 PMC4963827 27348002

[33] R. Ahmed , D. Gray , Science 1996, 272, 54. 8600537 10.1126/science.272.5258.54

[34] R. Kumar , P. Kumar , FEMS Yeast Res. 2019, 19, foz007. 30668686 10.1093/femsyr/foz007

[35] C. P. Karch , P. Burkhard , Biochem. Pharmacol. 2016, 120, 1. 27157411 10.1016/j.bcp.2016.05.001 PMC5079805

