# Supplementary material for: Safety and Immunogenicity of a Recombinant Tetanus Vaccine in Healthy Adults in China: A Randomized, Double‐Blind, Dose Escalation, Placebo‐ and Positive‐Controlled, Phase 1/2 Trial
Source: Adv Sci (Weinh). 2021 Jun 3;8(15):2002751. doi: 10.1002/advs.202002751 (PMC8336487; doi:10.1002/advs.202002751)
Supplement: Supplementary file 1 — Supporting Information [file ADVS-8-2002751-s001.pdf]

© 2021 Wiley-VCH GmbH

ADVANCED SCIENCE
Open Access

Supporting Information

for *Adv. Sci.,* DOI: 10.1002/advs.202002751

Safety and Immunogenicity of a Recombinant Tetanus Vaccine in Healthy Adults in China: a Randomized, Double-blind, Dose Escalation, Placebo- and Positive-Controlled, Phase 1/2 Trial

Xiaowei Xu, Rui Yu, Lanlan Xiao, Jie Wang, Meihong Yu, Junjie Xu, Yajun Tan, Xiao Ma, Xiaoxin Wu, Jiangshan Lian, Kaizhou Huang, Xiaoxi Ouyang, Sheng Bi, Shipo Wu, Xiaoyan Wang, Jiandi Jin, Ling Yu, Huafen Zhang, Qi Wei, Jinfa Shi, Wei Chen and Lanjuan Li**

Supporting Information

Inclusion/exclusion criteria

Inclusion criteria

1. Men or women aged >18 and <60 years
2. Ability to comprehend the full nature and purpose of the study, including possible risks and side effects; ability to co-operate with the investigator and to comply with the requirements of the entire study
3. Volunteers can comply with the requirements of the clinical trial program and complete all follow-up studies;
4. Ear temperature ≤ 37.5 degree C;
5. BMI index 19-32;
6. Signed informed consent obtained before any trial-related activities
7. Normal findings in the medical history, by physical examination and normal laboratory tests

Exclusion criteria for first vaccination

1. Women who are pregnant or breastfeeding, or who have a childbirth plan during the first three months of the study;
2. Infected with tetanus;
3. Accepted inoculation of tetanus vaccine in past 5 years, tetanus-diphtheria combined vaccine or polysaccharide-binding vaccine with TT as carrier protein;
4. allergies, such as those who are allergic to two or more drugs or food allergies, or are known to be allergic to the vaccine component (aluminum hydroxide adjuvant, sodium chloride);
5. Serious adverse reactions to any vaccine, such as allergies, urticaria, dyspnea, angioneurotic edema or abdominal pain;
6. Autoimmune diseases, immunodeficiency or HIV positive, important organs are primary diseases;
7. Systemic chemotherapy in the past 5 years, systemic immunosuppressive therapy, radiation therapy, cytotoxic therapy, high-dose inhaled corticosteroids in the past 6 months (excluding corticosteroid spray therapy for allergic rhinitis, acute non- Treatment of dermatitis with topical corticosteroids); prescription and/or non-prescription patients used within 2 months; physician-diagnosed coagulation disorders (such as clotting factor deficiency, coagulopathy, platelet abnormalities), or significant bruising or clotting disorders;
8. Received other study drugs within the first 3 months of receiving the vaccine for this study;
9. Received blood products within the first 3 months of receiving the vaccine for this study;
10. Live attenuated vaccines received within 30 days of receiving the vaccine for this study;
11. Received a subunit or inactivated vaccine, such as a pneumococcal vaccine, or anti-allergic therapy within the first 14 days of receiving the vaccine for this study;
12. Ongoing anti-TB prevention or treatment;
13. According to the researcher's judgment, due to various medical, psychological, social, occupational, or other conditions, it is contrary to the trial program or affects the subject's signing of informed consent.

Second vaccination exclusion criteria

If one of the following (1) to (3) adverse events (AEs) occurs, the continuation of vaccination is prohibited, but other study steps may be continued at the discretion of the investigator; if one of the following (4) to (5) adverse events occurs The investigator determines whether to continue the vaccination; if one of the following (6) to (7) adverse events occurs, the vaccination may be postponed within the time window set by the plan (without the occurrence of (6) to (7) adverse events. Two-day vaccination is on the 28th day, postponed vaccination within the time window means vaccination within 3 days after 28 days). For the following AEs, subjects must be followed up to the event resolution:

(1) any serious adverse event that has a causal relationship with the vaccination of the test vaccine;

(2) Serious allergic reactions or hypersensitivity after vaccination (including urticaria/rashes occurring within 30 minutes of vaccination);

(3) The occurrence of any first needle exclusion criteria after the election;

(4) Acute or new chronic diseases occurred during this vaccination;

(5) Grade 3 or above adverse reactions occurred within 72 hours of the previous vaccination;

(6) Acute disease at the time of vaccination (acute disease refers to moderate or severe disease with or without fever);

(7) Ear temperature >37.5°C at the time of vaccination.

Safety assessment

Vital sign checks including heart rate, blood pressure, respiratory rate, and ear temperature measurements were assessed at baseline. Blood samples for safety analysis were taken and assessed at baseline and at three predefined time points: 7 days, 28 days after first vaccination, and 7 days after second vaccination (**Table 1**). Participants had a telephone visit for safety analysis within 0–7 days after each vaccination.

Additionally, participants were required to keep a study diary to record any adverse events.

Safety monitoring was carried out by Clinical Research Coordinators at the study site.

Safety assessment at screening visit included:

• Medical history

• Concomitant medication

• Body weight and height

• Vital signs

• Hematology: red blood cell count (RBC), white blood cell count (WBC), leukocyte differential count (neutrophils, basophils, eosinophils, monocytes and lymphocytes), platelet count, hemoglobin, hematocrit

• Clinical chemistry (renal and hepatic function): Alanine transaminase (ALT), Aspartate transaminase (AST), albumin, Globulin, Bilirubin, total bile acid,
Blood glucose, Urea, creatinine, Total Cholesterol,
Triglyceride, High density liptein cholesterol, low density liptein cholesterol, blood urea nitrogen (BUN), potassium kalium, sodium, chlorine, phosphorus, calcium.

Urine: PH, proportion, color, turbidity, urine creatinine, RBC, WBC, cast, bacteria, epithelial cells, crystal.

- Pregnancy test

Supplementary Table 1. Visit contents and schedule

Item	Visit points	V0<br>-14～-1 day	V1<br>0 day	V2<br>V1+7 days (±1 day)	V3<br>V1+day (±2 days)	V4<br>V1+28 days (±3 days)	V5<br>V4+7 days (±1 day)	V6<br>V4+14 days (±2 days)	V7<br>V4+28 days (±3 days)	V8<br>V4+6 months (±3 days)
1	Screening, informed consent	●								
2	Demographic information	●								
3	Health examination									
	Vital signs (BP, HR, ear temperature)	●								
	Laboratory examination	●								
	Pregnancy test	●								
4	Initial inclusion/exclusion criteria	●								
5	Ear temperature measurement		●			●				
6	Inclusion/exclusion criteria		●			●				
7	Enrolled, assigned random number		●							
8	Blood collected		●	●	●	●	●	●	●	●
9	Vaccination		●			●				
10	Observation for 30 minutes		●			●				
11	Distribute diary card		●			●				
12	Check diary card			●	●	●	●	●	●	
13	Collect diary card					●			●	
14	Report serious adverse events		●	●	●	●	●	●	●	●
15	Record the vaccination and visit		●	●	●	●	●	●	●	●

Supplementary Table 2. Solicited adverse reactions and unsolicited adverse events in the seven days following administration of dose 1.

Recombinant tetanus vaccine							
	Placebo	TT vaccine	Low-dose	Medium-dose	High-dose	All doses combined	p-value*
	(N=30)	(N=30)	(N=30)	(N=30)	(N=30)	(N=90)	
**Solicited adverse reactions within 0–7 days**							
**Any**	13	10	8	12	12	32	0·6814
Grade 1	12	9	8	10	6	24	0·529
Grade 2	1	1	0	1	5	6	0·0839
Grade 3	0	0	0	1	1	2	1
**Injection-site adverse reactions**							
Pain							
Grade 1	7	6	4	7	6	17	0·8698
Grade 2	1	0	0	0	0	0	1
Induration							
Grade 1	7	1	2	4	2	8	0·142
Grade 2	0	0	0	1	1	2	1
Redness							
Grade 1	6	1	0	8	5	13	0·0041

Grade 2	0	1	0	0	4	4	0·0283
Grade 3	0	0	0	1	1	2	1
Itch							
Grade 1	1	1	0	1	3	4	0·522
Grade 2	0	0	0	1	0	1	1
Rash							
Grade 2	0	0	0	0	1	1	1
**Systemic adverse reactions**							
Fever							
Grade 1	0	0	0	0	0	0	1
Fatigue							
Grade 1	0	0	0	0	0	0	1
Nausea							
Grade 1	0	0	0	1	0	1	1
Cough							
Grade 1	0	1	0	0	0	0	1
Pain							
Grade 1	0	0	1	1	0	2	1
Pharyngeal diseases							
Grade 1	0	1	0	0	0	0	1

Headache							
Grade 1	0	1	0	0	0	0	1

Supplementary Table 3. Solicited adverse reactions and unsolicited adverse events in the seven days following administration of dose 2.

Recombinant tetanus vaccine							
	Placebo	TT vaccine	Low-dose	Medium-dose	High-dose	All doses combined	p-value*
	(N=30)	(N=30)	(N=30)	(N=30)	(N=30)	(N=90)	
**Solicited adverse reactions within 0–7 days**							
**Any**	10	17	14	14	14	42	0.4973
Grade 1	6	14	12	12	8	32	0.1627
Grade 2	4	3	2	2	4	8	0.8973
Grade 3	0	0	0	0	2	2	0.1958
**Injection-site adverse reactions**							
Pain							
Grade 1	5	5	10	6	6	22	0.4324
Induration							
Grade 1	3	5	6	7	3	16	0.5463
Grade 2	0	3	0	2	1	3	0.3148

Redness							
Grade 1	3	2	4	7	4	15	0.4774
Grade 2	4	0	1	1	2	4	0.2547
Grade 3	0	0	0	0	2	2	0.1958
Itch							
Grade 1	0	1	0	3	3	6	0.1511
Rash							
Grade 2	0	1	0	0	0	0	1
**Systemic adverse reactions**							
Fever							
Grade 1	0	0	1	0	0	1	0.1869
Fatigue							
Grade 1	1	0	0	0	0	0	1
Nausea							
Grade 1	0	0	0	0	0	1	1
Cough							
Grade 1	0	1	0	0	0	0	1
Pain							
Grade 1	0	0	0	1	0	1	1
Pharyngeal diseases							

Grade 1	0	0	0	0	1	1	1
Headache							
Grade 1	0	0	0	0	0	0	1

The detail of six serious adverse events

Three cases were reported in two participants from placebo group, including two cases of knee injury and one case of acute carbon monoxide poisoning. The other three cases occurred in the medium-dose vaccine group, including one case who had a spontaneous abortion 3 months after second vaccination, one case who reported 5 months after second vaccination that she was pregnant and had an abortion five days after diagnosis of pregnancy, and one case who had anal fissure. Unfortunately, one participant had suffered from acute carbon monoxide poisoning and died.

Supplementary Table 4. **Proportion of seroconversion by anti-TT IgG antibody at each visit.**

	Placebo (n=28)	Positive vaccine (n=29)	Recombinant tetanus vaccine				p-value*
			Low-dose (n=27)	Medium-dose (n=30)	High-dose (n=28)	All doses combined (n=85)	
Day 7	2 (6.9)	7 (25.0)	8 (29.6)	3 (10.0)	7 (25.0)	18 (21.2)	0.1062
Day 14	1 (3.4)	23 (82.1)	21 (77.8)	18 (60.0)	20 (71.4)	59 (69.4)	<0.001
Day 28	1 (3.4)	26 (92.9)	24 (88.9)	19 (63.3)	26 (92.9)	69 (81.2)	<0.001
Day 35	3 (10.3)	27 (96.4)	27 (100.0)	30 (100.0)	27 (96.4)	84 (98.8)	<0.001
Day 42	2 (6.9)	28 (100.0)	27 (100.0)	30 (100.0)	27 (96.4)	84 (98.8)	<0.001
Day 56	2 (6.9)	28 (100.0)	27 (100.0)	30 (100.0)	28 (100.0)	85 (100.0)	<0.001
Day 208	1 (3.4)	27 (96.4)	27 (100.0)	29 (96.7)	27 (96.4)	83 (97.6)	<0.001

Data are n (%). *p-values were generated from comparisons across the five groups.

Supplementary Table 5. Proportion of seroconversion by anti-TeNT-Hc IgG antibody at each visit.

	Placebo (n=28)	TT vaccine (n=29)	Recombinant tetanus vaccine				p-value*
			Low-dose (n=27)	Medium-dose (n=30)	High-dose (n=28)	All doses combined (n=85)	
Day 7	0	4 (14.3)	1 (3.7)	1 (3.3)	9 (32.1)	11 (12.9)	0.0004
Day 14	4 (13.8)	14 (50.0)	17 (63.0)	13 (43.3)	13 (46.4)	43 (50.6)	0.0043
Day 28	3 (10.3)	20 (71.4)	23 (85.2)	25 (83.3)	26 (92.9)	74 (87.1)	<0.001
Day 35	10 (34.5)	25 (89.3)	27 (100.0)	30 (100.0)	27 (96.4)	84 (98.8)	<0.001
Day 42	7 (24.1)	25 (89.3)	27 (100.0)	30 (100.0)	27 (96.4)	84 (98.8)	<0.001
Day 56	7 (24.1)	25 (89.3)	26 (96.3)	30 (100.0)	26 (92.9)	82 (96.5)	<0.001
Day 208	6 (20.7)	18 (64.3)	26 (96.3)	29 (96.7)	24 (85.7)	79 (92.9)	<0.001

Data are n (%).

*p-values were generated from comparisons across the five groups.

Supplementary Table 6. Geometric mean titre of anti-TT IgG antibody, after initial and boost vaccinations.

			Recombinant tetanus vaccine				
Days	Placebo (N=29)	TT vaccine (N=28)	Low-dose (N=27)	Medium-dose (N=30)	High-dose (N=28)	All doses combined (N=85)	p-value*
Day 0	335.7 (247.0,456.1)	320.0 (241.1,424.8)	296.3 (222.2,395.1)	320.0 (239.8,427.0)	487.4(373.2,636.7)	358.7(304.8,422.2)	0.1082
Day 7	416.2 (307.6,563.2)	724.3 (502.2,1044.6)	534.7 (372.5,767.6)	432.1 (319.6,584.2)	724.3 (498.6,1052.2)	548.1(450.5,667.0)	0.0368
Day 14	429.7 (396.0,466.3)	4874.4 (2801.3,8481.7)	2068.3 (1343.8,3183.3)	2211.1 (1263.7,3868.5)	4638.9 (2677.0,8038.5)	2763.1 (2048.8,3726.5)	< 0.0001§†
Day 28	496.0 (423.0,581.6)	7801.7 (5196.5,11712.9)	2814.5 (1923.7,4117.8)	2539.8 (1504.6,4287.3)	9051.0 (5926.8,13822.0)	3988.1 (3014.5,5276.3)	< 0.0001§†
Day 35	709.9 (574.2,877.7)	14849.7(9923.8,22220 .6)	6912.4 (5236.3,9124.9)	7351.7 (4698.0,11504.2)	8829.7 (5975.8,13046.4)	7657.6 (6181.6,9486.1)	< 0.0001§†
Day 42	615.0 (501.3,754.5)	14849.7 (11046.6,19962.0)	9901.9 (7596.3,12907.2)	12222.0 (8374.5,17837.0)	11309.8 (8085.1,15820.7)	11143.0 (9254.5,13417.0)	< 0.0001§‡
Day 56	693.1 (565.2,850.0)	15603.4 (11262.9,21616.5)	10159.4 (7292.9,14152.4)	14039.4 (10226.6,19273.6)	17227.5 (12693.9,23380.4)	13551.9 (11297.3,16256.4)	< 0.0001§
Day 208	615.0 (501.3,754.5)	6893.4 (4518.5,10516.4)	6566.4 (4615.8,9341.3)	6859.4 (4807.3,9787.3)	7243.3 (5137.4,10212.4)	6887.4 (5670.5,8365.5)	< 0.0001§

Data are geometric mean titre (95% CI). Analysis of variance was used for the log-transformed antibody titres;

*p values were generated from then comparison across five groups (placebo, TT vaccine, low-dose, medium-dose, high-dose).
†Both the low-dose and medium-dose groups showed lower GMT than did TT group.
‡The low-dose groups showed lower GMT than did TT group.
§The high-dose, medium-dose and low-dose groups showed higher GMT than did the placebo group.

Supplementary Table 7. Geometric mean titre of anti-TeNT-Hc IgG antibody, after initial and boost vaccinations.

			Recombinant tetanus vaccine				
Days	Placebo (N=29)	TT vaccine (N=28)	Low-dose (N=27)	Medium-dose (N=30)	High-dose (N=28)	All doses combined (N=85)	p-value*
Day 0	416.2(298.2,581.1)	399.9(264.3,605.0)	354.6(248.8,505.4)	508.0(345.1,747.6)	656.0(435.2,989.0)	493.0(395.6,614.3)	0.1649
Day 7	387.4(278.6,538.8)	551.7(361.6,841.7)	383.0(264.7,554.1)	508.0(353.8,729.4)	1448.7(1071.0,1959.6)	655.9(522.3,823.6)	< 0.0001†
Day 14	508.0(425.8,606.1)	1810.8(1246.6,2630.5)	2412.7(1610.4,3614.8)	2315.6(1394.1,3846.3)	4098.8(2479.5,6775.7)	2831.6(2161.1,3710.0)	< 0.0001§†
Day 28	676.7(537.7,851.8)	3621.6(2409.2,5444.3)	3456.2(2452.8,4870.0)	5079.7(3410.9,7564.9)	13449.7(9702.6,18643.8)	6194.6(4902.1,7828.0)	< 0.0001§†
Day 35	1145.0(882.8,1485.0)	6243.5(4448.2,8763.5)	11550.8(8716.6,15306.5)	14039.4(10081.8,19550.4)	14486.6(10806.3,19420.2)	13332.7(11247.4,15804.6)	< 0.0001§‡
Day 42	945.7(743.5,1202.9)	6724.8(5169.5,8748.1)	16127.0(13005.8,19997.2)	18101.9(13382.5,24485.7)	20487.1(15282.4,27464.4)	18175.9(15580.0,21204.3)	< 0.0001§‡
Day 56	800.0(566.5,1129.8)	4201.6(3185.0,5542.6)	12159.4(9350.9,15811.4)	15758.6(11362.9,21855.0)	13786.8(9994.3,19018.3)	13887.5(11696.2,16489.4)	< 0.0001§‡
Day 208	744.6(593.6,934.1)	2971.0(2230.9,3956.6)	7659.9(5518.4,10632.6)	8252.0(6113.1,11139.3)	8613.8(6462.6,11481.0)	8173.8(6902.4,9679.4)	< 0.0001§‡

Data are geometric mean titre (95% CI). Analysis of variance was used for the log-transformed antibody titres;
*p values were generated from then comparison across five groups (placebo, TT vaccine, low-dose, medium-dose, high-dose).
†The high-dose groups showed higher GMT than did TT group.
‡ The high-dose, medium-dose and low-dose groups showed higher GMT than did TT group.
§The high-dose, medium-dose and low-dose groups showed higher GMT than did the placebo group.

Supplementary Table 8. Geometric mean concentration of anti-TT IgG antibody, after initial and boost vaccination (IU/mL).

			Recombinant tetanus vaccine				
Days	Placebo (N=29)	TT vaccine (N=28)	Low-dose (N=27)	Medium-dose (N=30)	High-dose (N=28)	All doses combined (N=85)	p-value*
Day 7	0.0809±0.0420	0.1303±0.0863	0.0833±0.0519	0.1114±0.0577	0.1426±0.0778	0.1128±0.0671	0.0008
Day 14	0.3710±0.3301	3.6420±2.0254	2.3458±1.3598	2.5794±1.6504	3.0060±2.1572	2.6457±1.7556	< 0.0001§ ‡
Day 28	0.3310±0.2983	3.9891±1.6241	2.3686±1.1626	2.7035±1.5355	2.5706±1.7954	2.5533±1.5125	< 0.0001§†
Day 35	0.3661±0.2921	3.7812±1.5433	2.1044±0.9897	2.8214±1.5803	2.9706±1.5045	2.6428±1.4259	< 0.0001§ ‡
Day 42	0.3616±0.3663	3.8526±1.1293	3.1462±1.0514	3.4574±1.4293	3.5800±1.2352	3.3989±1.2532	< 0.0001§
Day 56	0.4968±0.4010	3.9588±0.9956	3.5495±0.9061	3.8840±1.8091	3.8699±1.2423	3.7731±1.3797	< 0.0001§
Day 208	0.2103±0.2320	1.8479±1.3180	1.5421±0.8201	1.9055±1.4107	1.7749±1.3473	1.7470±1.2253	< 0.0001§

Data are geometric concentration ± SD. Analysis of variance was used for the log-transformed antibody titres.

*p-values were generated from comparisons across the five groups (placebo, positive vaccine, low-dose, medium-dose, high-dose).

†The high-dose, medium-dose, and low-dose groups showed lower GMC than did the positive group.

‡The medium-dose and low-dose groups showed lower GMC than did the positive group.

§The high-dose, medium-dose, and low-dose groups showed higher GMC than did the placebo group.

Supplementary Table 9. Geometric mean concentration of anti-TeNT-Hc IgG antibody, after initial and boost vaccination.

Days	Placebo	TT vaccine	Low-dose	Medium-dose	High-dose	All doses combined	p value*
	(N=29)	(N=28)	(N=27)	(N=30)	(N=28)	(N=85)	
Day 7	0.4121±0.3047	0.5329±0.3581	0.4990±0.2557	0.6112±0.3310	0.6823±0.5133	0.5990±0.3851	0.0585
Day 14	1.2137±1.0429	7.7926±5.1995	9.0200±6.2999	10.5414±8.0957	13.1353±9.1148	10.9126±8.0289	< 0.0001§$
Day 28	1.4292±1.5697	6.8944±4.8044	9.1239±7.3395	11.2247±8.0332	11.5039±9.3512	10.6494±8.2565	< 0.0001§‡
Day 35	1.3618±1.3421	7.1498±4.7101	9.9900±5.8722	12.3425±7.6588	15.0076±6.8477	12.4732±7.0799	< 0.0001§‡
Day 42	1.3454±1.3221	7.9820±4.7087	12.5577±4.3765	14.7050±7.6287	15.3768±5.4663	14.2442±6.0863	< 0.0001§†
Day 56	1.5943±1.4170	8.7039±3.9108	14.3883±5.5136	15.7401±7.4260	16.1909±6.1032	15.4592±6.4028	< 0.0001§†
Day 208	1.0426±0.8659	4.3914±3.1271	8.6911±6.0057	10.0509±9.3008	10.6525±5.1227	9.8171±7.0798	< 0.0001§†

Data are geometric concentration ± SD. Analysis of variance was used for the log-transformed antibody titres.

*p values were generated from comparisons across the five groups (placebo, positive vaccine, low-dose, medium-dose, high-dose).

†The high-dose, medium-dose, and low-dose groups showed higher GMC than did the positive group.

‡The medium-dose and high-dose groups showed higher GMC than did the positive group.

$The high-dose group showed higher GMC than did the positive group.

§The high-dose, medium-dose, and low-dose groups showed higher GMC than did the placebo group.

Supplementary Table 10. Interleukin-2 concentrations

			Recombinant tetanus vaccine				
Days	Placebo (N=29)	TT vaccine (N=28)	Low-dose (N=27)	Medium-dose (N=30)	High-dose (N=28)	All doses combined (N=85)	p-value*
Day 0	3.0 (0.0, 5.0)	0.0 (0.0, 6.5)	0.0 (0.0, 3.0)	3.0 (0.0, 20.0)	4.0 (0.0, 14.0)	0.0 (0.0, 13.0)	0.0957
Day 7	3.0 (0.0, 15.0)	9.0 (1.5, 29.0)	13.0 (0.0, 25.0)	11.5 (0.0, 28.0)	26.5 (4.0, 54.0)	15.0 (0.0, 38.0)	0.3947
Day 14	10.0 (0.0, 28.0)	52.5 (8.0, 80.5)	83.0 (25.0, 143.0)	79.0 (43.0, 190.0)	61.5 (6.5, 190.0)	78.0 (25.0, 155.0)	0.0202§†
Day 28	0.0 (0.0, 3.0)	33.0 (9.0, 63.0)	115.0 (65.0, 218.0)	80.5 (30.0, 245.0)	70.0 (40.0, 141.5)	88.0 (40.0, 210.0)	0.0001§‡
Day 35	3.0 (0.0, 10.0)	30.5 (14.0, 65.5)	140.0 (75.0, 250.0)	78.0 (38.0, 130.0)	98.0 (47.5, 181.5)	100.0 (48.0, 175.0)	< 0.0001§†
Day 42	0.0 (0.0, 5.0)	44.0 (19.0, 81.5)	145.0 (70.0, 325.0)	167.5 (70.0, 285.0)	204.0 (78.0, 347.5)	180.0 (70.0, 325.0)	< 0.0001§‡
Day 56	0.0 (0.0, 5.0)	42.5 (16.5, 65.0)	215.0 (88.0, 328.0)	147.5 (60.0, 308.0)	139.0 (59.0, 234.0)	158.0 (65.0, 285.0)	< 0.0001§‡
Day 208	5.0 (0.0, 8.0)	19.0 (9.0, 40.5)	103.0 (48.0, 230.0)	121.5 (68.0, 218.0)	173.0 (96.5, 250.0)	133.0 (68.0, 230.0)	< 0.0001§‡

Data are median (Q1, Q3).

*p values were generated from comparisons across the five groups (placebo, positive vaccine, low-dose, medium-dose, high-dose).

†The low-dose and high-dose groups showed higher interleukin-2 concentrations than did the positive group.

‡The high-dose, medium-dose, and low-dose groups showed higher interleukin-2 than did the positive group.

§The high-dose, medium-dose, and low-dose groups showed higher interleukin-2 than did the placebo group.

Supplementary Table 11. Interferon-γ concentrations

			Recombinant tetanus vaccine				
Days	Placebo	TT vaccine	Low-dose	Medium-dose	High-dose	All doses combined	p-value*
	(N=29)	(N=28)	(N=27)	(N=30)	(N=28)	(N=85)	
Day 0	0.0 (0.0, 3.0)	0.0 (0.0, 4.0)	3.0 (0.0, 8.0)	0.0 (0.0, 3.0)	0.0 (0.0, 3.0)	0.0 (0.0, 3.0)	0.9278
Day 7	0.0 (0.0, 3.0)	3.0 (0.0, 6.5)	0.0 (0.0, 18.0)	0.0 (0.0, 3.0)	14.0 (0.0, 64.0)	3.0 (0.0, 20.0)	0.0781$
Day 14	3.0 (0.0, 5.0)	11.5 (0.0, 37.5)	25.0 (0.0, 98.0)	10.0 (0.0, 53.0)	33.0 (10.0, 79.0)	18.0 (3.0, 73.0)	0.2599
Day 28	0.0 (0.0, 3.0)	8.0 (0.0, 16.5)	20.0 (3.0, 35.0)	11.5 (0.0, 35.0)	10.0 (1.5, 30.0)	14.0 (0.0, 33.0)	0.0561§
Day 35	3.0 (0.0, 5.0)	9.0 (3.0, 29.0)	38.0 (10.0, 95.0)	30.0 (8.0, 68.0)	25.5 (3.0, 75.5)	30.0 (5.0, 75.0)	0.0166§†
Day 42	3.0 (0.0, 8.0)	9.0 (0.0, 20.5)	60.0 (18.0, 135.0)	30.5 (8.0, 90.0)	31.5 (10.5, 161.5)	38.0 (8.0, 133.0)	0.0122§†$
Day 56	0.0 (0.0, 10.0)	4.0 (0.0, 16.5)	28.0 (15.0, 78.0)	24.0 (8.0, 50.0)	20.5 (8.0, 54.0)	25.0 (8.0, 53.0)	0.0001§‡
Day 208	0.0 (0.0, 10.0)	1.5 (0.0, 8.0)	25.0 (0.0, 100.0)	39.0 (8.0, 68.0)	21.5 (0.0, 61.5)	27.0 (0.0, 65.0)	0.0015§‡

Data are median (Q1, Q3).

*p-values were generated from comparisons across the five groups (placebo, positive vaccine, low-dose, medium-dose, high-dose).

†The low-dose group showed higher interferon-γ concentrations than did the positive group.

$The high-dose group showed higher interferon-γ than did the positive group.

‡ The high-dose, medium-dose, and low-dose groups showed higher interferon-γ than did the positive vaccine group.

§The high-dose, medium-dose, and low-dose groups showed higher interferon-γ than did the placebo group.
